# Long-Term Outcomes after Vaccine-Induced Thrombotic Thrombocytopenia

**DOI:** 10.3390/v14081702

**Published:** 2022-08-01

**Authors:** Victoria Panagiota, Christiane Dobbelstein, Sonja Werwitzke, Arnold Ganser, Nina Cooper, Ulrich J. Sachs, Andreas Tiede

**Affiliations:** 1 Hematology, Hemostasis, Oncology and Stem Cell Transplantation, Hannover Medical School, 30625 Hannover, Germany; panagiota.victoria@mh-hannover.de (V.P.); dobbelstein.christiane@mh-hannover.de (C.D.); werwitzke.sonja@mh-hannover.de (S.W.); ganser.arnold@mh-hannover.de (A.G.); 2 Department of Thrombosis and Hemostasis, Giessen University Hospital, Giessen, Germany, and Institute for Clinical Immunology and Transfusion Medicine, Justus Liebig University, 35390 Giessen, Germany; nina.cooper@immunologie.med.uni-giessen.de

**Keywords:** AZD1222, ChAdOx1 nCoV-19, COVID-19, SARS-CoV-2, vaccination, platelet activation, thrombosis, cerebral venous sinus thrombosis, cohort studies

## Abstract

Vaccine-induced thrombotic thrombocytopenia (VITT), or thrombosis with thrombocytopenia syndrome (TTS), is a rare but serious complication of adenovirus-based vaccines against severe respiratory syndrome coronavirus 2 (SARS-CoV-2). Observation of long-term outcomes is important to guide treatment of affected patients. This single-center consecutive cohort study included all patients diagnosed based on (1) vaccination 4 to 21 days before symptom onset, (2) signs or symptoms of venous or arterial thrombosis, (3) thrombocytopenia < 150/nL, (4) positive anti-platelet factor 4 (PF4) antibody, and (5) elevated D-Dimer > 4 times the upper limit of normal. Nine patients were enrolled. Acute management consisted of parenteral anticoagulants, corticosteroids, intravenous immunoglobulin (IVIG), and/or eculizumab. Eculizumab was successfully used in two patients with recurrent thromboembolic events after IVIG. Direct oral anticoagulants were given after hospital discharge. Median follow-up duration was 300 days (range 153 to 380). All patients survived the acute phase of the disease and were discharged from hospital. One patient died from long-term neurological sequelae of cerebral venous sinus thrombosis 335 days after diagnosis. Eight out of nine patients were alive at last follow-up, and seven had fully recovered. Anti-PF4 antibodies remained detectable for at least 12 weeks after diagnosis, and D-Dimer remained elevated in some patients despite oral anticoagulation. No recurrent thromboembolic events, other signs of VITT relapse, or bleeding complications occurred after discharge. In conclusion, VITT appears to be a highly prothrombotic condition. IVIG is not always successful, and eculizumab may be considered a rescue agent. Long-term management with direct oral anticoagulants appears to be safe and effective.

## 1. Introduction

Vaccines against coronavirus disease (COVID-19) were developed, licensed, and rolled out for use in the general population with unprecedented speed and success [1]. Since the beginning of 2021, about five billion people have received about 13 billion inoculations [2]. Never has a newly licensed medicine been used by more people in such a short period of time. This may have contributed to the early discovery of a new syndrome, called vaccine-induced thrombotic thrombocytopenia (VITT) or thrombosis with thrombocytopenia syndrome (TTS) [3,4].

VITT/TTS occurs 4 to 30 days after vaccination with recombinant adenoviral vectors encoding the SARS-CoV-2 spike protein, i.e., Vaxzevria (AZD1222) or Jcovden (Ad26.COV2-S) [5,6]. The pathology of the syndrome involves formation of autoantibodies against platelet factor 4 (PF4) that activate platelets, leading to thrombocytopenia and thromboembolic events of varying severity and location [7]. Sharing striking similarities with heparin-induced thrombocytopenia (HIT), TTS/VITT was soon recommended to be managed similarly: avoiding heparins and using alternative anticoagulation (e.g., with argatroban) [8,9]. High-dose intravenous immunoglobulin (IVIG) has also been recommended in analogy to treatment-refractory HIT cases and the rare autoimmune form of HIT that occurs without prior exposure to heparin [10].

Here, we report one-year outcomes of consecutive patients with VITT/TTS diagnosed at Hannover Medical School. Together with similar reports from other institutions, our data confirm that this syndrome is a highly thrombogenic disorder. Our data also shed light on the risk of failure of IVIG and on using eculizumab as an alternative mode of treatment.

## 2. Materials and Methods

We report results of a retrospective, consecutive cohort study of patients diagnosed with VITT/TTS based on the presence of all of the following criteria:

- Vaccination 4 to 21 days before symptom onset.
- Signs or symptoms of venous or arterial thrombosis.
- Thrombocytopenia < 150/nL.
- Positive anti-platelet factor 4 (PF4) antibody.
- Elevated D-Dimer > 4 times upper limit of normal.

The initial course of 5 of these patients has been reported previously [7]. There was no prespecified treatment protocol at the time. However, the German, Austrian and Swiss Society on Thrombosis and Haemostasis (GTH) ad hoc recommendations on diagnosis and management of VITT were used to guide treatment as soon as published (end of March 2021) [9]. After discharge from our hospital, most patients were followed in our outpatient clinic except for patient 5, for whom data were obtained by consulting the rehabilitation clinic.

We collected baseline clinical and demographic data; details on thromboembolic events and bleeding; treatment information with regards to anticoagulation, immunosuppression, use of intravenous immunoglobulin (IVIG), and eculizumab; and mortality and status of neurological and general health recovery at study end.

Anti-PF4 autoantibodies were assessed as previously reported [7]. In brief, anti-PF4 IgG was detected with enzyme-linked immunosorbent assay (ELISA, Hyphen BioMed, CoaChrom, Maria Enzersdorf, Austria). Flow cytometry of patient immunoglobulin (Ig) binding to healthy donor platelets was performed using platelets from type 0 donors, freshly isolated and pre-incubated with saline or AZD1222 in the absence or presence of heparin (100 IU/mL) for 30 min. Serum samples were subsequently added. After 30 min, fluorescein isothiocyanate-labelled anti-human immunoglobulin G was added to detect antibody binding. The drug-to-saline mean fluorescence intensity (MFI) ratio was calculated. Ratios above 2.0 were considered positive. Heparin-induced platelet aggregation (HIPA) tests were performed with washed platelets from four different donors in the absence (buffer alone) or presence of heparin (0.2 or 100 IU/mL). For the modified test, AZD1222 was added. Reactions were placed in microtiter wells containing spherical stir bars and stirred at approximately 500 rpm. Wells were visually examined in 5 min intervals for loss of turbidity. Sera were interpreted as reactive (positive) if a shift from turbidity to transparency occurred within 40 min in at least two suspensions. Each test included positive and negative control sera.

## 3. Results

### 3.1. Presenting Characteristics

Between 8 March and 11 June 2021, nine patients were diagnosed (Table 1). Eight of them were female, and all but one were diagnosed after the first vaccination. At the time of diagnosis, anti-PF4 antibodies were highly positive using ELISA but negative using the chemiluminescence immunoassay (CLIA) [11]. HIPA was positive with buffer alone in four out of nine patients, with low-dose heparin (0.2 IU/mL) in two of nine patients, and with high-dose heparin (100 IU/mL) in zero out of nine patients. The modified HIPA with addition of AZD1222 was positive in seven out of nine patients. Flow cytometry detected binding of patient Ig to healthy platelets in all tested patients (data missing in patient 9). Thromboembolic events were confirmed by imaging studies in eight out of nine patients. Six patients also had signs of bleeding, including petechiae in all of them, and secondary bleeding into areas of cerebral infarction in two patients.

### 3.2. Details on Thromboembolic Events

All but one patient had a confirmed thromboembolic event. Details are listed in Table 2. Details on events in patients 1 to 5 have previously been reported [7]. The spectrum of clinical events was broad ranging from transitory ischemia attack to life-threatening CVST and arterial infarctions. 

Venous thrombotic or embolic events were diagnosed in six patients. Three of them had venous thrombosis in unusual sites (cerebral sinus [pat. 1], splanchnic veins [pat. 4], and portal vein [pat. 8]). Arterial events were present in three patients (including an embolic cerebral artery occlusion [pat. 2], TIA [pat. 3], and thrombotic occlusions of cerebral and popliteal arteries [pat. 5]). One patient had evidence of thrombotic microangiopathy (pat. 1).

The only patient without a confirmed thromboembolic event (pat. 7) presented with severe headache and suspicion of CVST in initial imaging results but was later judged to have had aplasia of the right rectal sinus rather than thrombosis.

### 3.3. Initial Therapy

All patients received full-dose anticoagulation as an initial therapy. Patient 1 received unfractionated heparin (UFH) because the diagnosis was initially unclear and recommendations for using argatroban were not yet available. All other patients received argatroban for initial anticoagulation. Platelet infusion was avoided despite low platelet counts.

Corticosteroids were given in six patients, usually with prednisolone 100 mg per day for 3 to 5 days. 

IVIG was used as first-line treatment in four patients (1 g/kg on days one and two). Two of those patients had no further events and were switched to oral anticoagulation. The other two patients had subsequent events (pat. 4, splanchnic vein thrombosis; pat. 6, new DVT). These patients received eculizumab (900 mg i.v.) as a second-line therapy, resulting in fast improvement and resolution of VITT pathology.

Eculizumab was also given in pat. 1 as a first-line treatment because of clinical and laboratory signs of thrombotic microangiopathy (TMA). It resulted in prompt resolution of TMA and signs of VITT.

### 3.4. Long-Term Therapy

**Figure 1:**
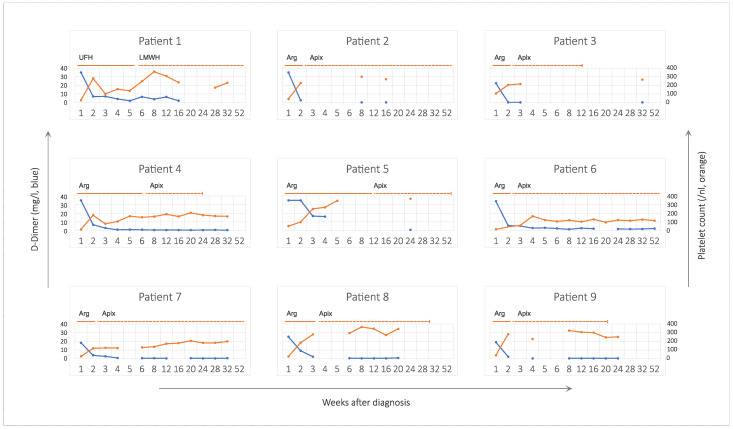
D-Dimer and platelet count for individual patients over time. Blue lines indicate D-Dimer (left axis, linear, reference value < 0.5 mg/L) and orange lines indicate platelet count (right axis, reference range 150–450 /nL). Duration of anticoagulation is indicated by solid and dashed lines for parenteral and oral anticoagulants, respectively. Abbreviations: AF, atrial fibrillation; Apix, apixaban; Arg, argatroban; LMWH, low molecular weight heparin; UFH, unfractionated heparin.

Parenteral anticoagulation was switched to oral apixaban, when the platelets had returned to normal, and imaging indicated stabilization of the initial thromboembolic event (Figure 1), usually after 2 weeks. The exceptions were patients 1 and 5, in whom oral administration was impossible due to neurological status, and patient 4, who still suffered from critical splanchnic vein thrombosis and was switched to oral administration in week six.

IVIG was not continued after the initial cycle of 2 days. Eculizumab was continued in patients 1 and 4 until week eight and in patient 6 until week seven (weeks one to four, 900 mg weekly, and then 1200 mg biweekly).

Long-term anticoagulation was maintained for 6 to 12 months. Patients 1, 2, and 6 had atrial fibrillation requiring indefinite anticoagulation. Patients 3 and 9 stopped anticoagulation already after 12 and 20 weeks, respectively, because the clinical course was mild, D-Dimer was normal, and imaging indicated complete resolution of thromboembolic events.

### 3.5. Platelet Count and D-Dimer

Platelet counts recovered within one week except for patients 6 and 7, in whom platelets recovered to >150/nL in week 4 and 12, respectively (Figure 1). There was no relapse of thrombocytopenia after initial therapy. The course of platelet counts was not grossly different between patients receiving anticoagulation alone and those receiving additional therapies such as corticosteroids, IVIG, or eculizumab.

**Figure 2:**
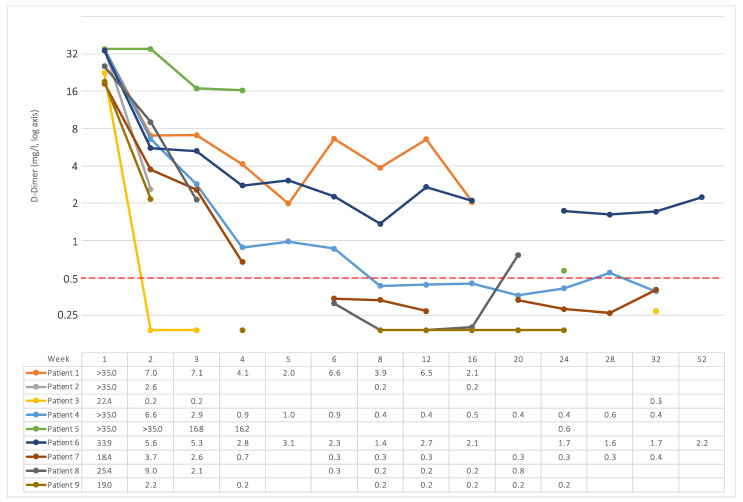
D-Dimer for individual patients on logarithmic axis. The dashed red line indicates the upper limit of normal (0.5 mg/L). Individual results are shown in the table below the figure in mg/L.

D-Dimer was initially very high (>18 mg/L in all patients). It usually decreased steeply after starting parenteral anticoagulation but remained at least slightly elevated in most patients for several months (Figure 1). Figure 2 shows individual courses of D-Dimer on a logarithmic scale to highlight the duration of elevation above the reverence range.

### 3.6. Anti-PF4 Antibodies and Platelet Binding

**Figure 3:**
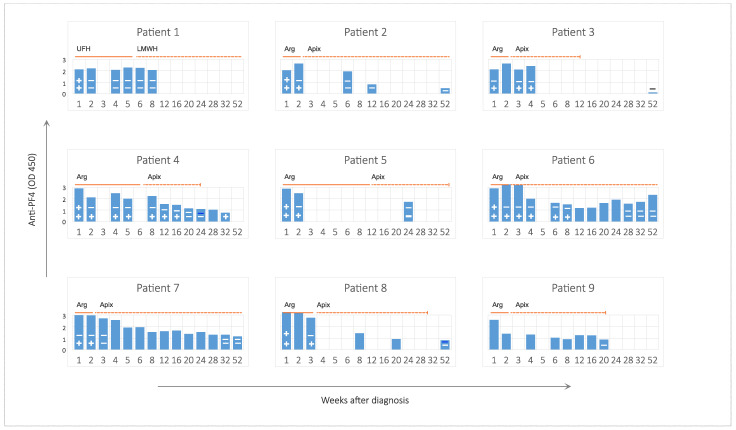
Anti-PF4 IgG (ELISA) and binding of patient Ig to normal platelets (flow cytometry) for individual patients over time. Blue bars indicate anti-PF4 ELISA (in arbitrary units at OD450, reference value < 0.36). “+” and “−” signs in the bars indicate positive and negative results for platelet aggregation (HIPA, top row) or binding (flow cytometry, bottom row) of patient Ig to normal platelets, respectively. Duration of anticoagulation is indicated by solid and dashed lines for parenteral and oral anticoagulants, respectively. Abbreviations: AF, atrial fibrillation; Apix, apixaban; Arg, argatroban; LMWH, low molecular weight heparin; UFH, unfractionated heparin.

A detailed description of anti-PF4 antibody findings at baseline has been published for the initial five patients [7]. Because the chemiluminescent assay was non-reactive in VITT patients, we used the ELISA to observe the time course of autoantibodies (Figure 3). ELISA-detectable anti-PF4 antibodies did not become negative before week 12. In two patients, they were detectable for at least 1 year (Figure 3). 

Platelet-aggregating and binding potential of antibodies disappeared much earlier: by week two, aggregation in the HIPA test was negative in all tested patients; by week four, all but two were negative for platelet binding of patient Ig (Figure 3). In one patient [pat. 4], platelet-binding antibodies were detectable again in week 32, when ELISA antibodies were already <1 OD. 

There was no obvious correlation between the duration of platelet binding or aggregating antibody detection and platelet count recovery. Likewise, anti-PF4 antibodies detected in ELISA or functional studies did not correlate with the levels of D-Dimer.

### 3.7. Outcome

Median clinical follow-up was 300 days (range, 153 to 380). Patients 1 and 5 received tracheotomy and were transferred to intensive care rehabilitation after discharge from the university hospital. Patient 1 died on day 335 from neurological sequelae of CVST. Patient 5 recovered with neurological sequelae of cerebral infarction (modified Rankin scale 5). All other patients were sent home from the university hospital, were followed as outpatients in our clinic, and recovered without sequelae. They received their second COVID-19 vaccination with an mRNA vaccine without complications a mean of 255 days (range 125–494) after the initial vaccination.

## 4. Discussion

This case series confirms that using direct parenteral and oral anticoagulants is safe and effective in VITT/TTS. Despite detectable anti-PF4 antibodies and elevated D-Dimer in most of our patients, thromboembolic events were clinically under control. We did not see unusual bleeding events despite low platelet counts while using these anticoagulants. Heparin was used in only one patient of this series, and it is difficult to say whether this anticoagulant is generally safe in VITT/TTS.

Our series sheds some light on the effectiveness and safety of IVIG. In accordance with other reports [3,4,12,13], we saw increasing platelet counts in all patients after IVIG. However, most of our patients did not receive IVIG, and their platelet counts recovered equally well. We also witnessed new thromboembolic events in two out of four treated patients. These occurred while on anticoagulation and despite increasing platelets, which are usually considered a sign of recovery. We used eculizumab in these cases, because it had already resolved the VITT/TTS pathology in our first patient, who had been treated before ad hoc recommendations from experts had become available. Eculizumab resulted in clinical resolution of thromboembolic events with no further events being observed.

The pathogenesis of VITT/TTS is not entirely clear but seems to involve activation of platelets by anti-PF4 antibodies that were elicited by vaccination. It has long been known that low-positive anti-PF4 antibodies occur in the general population and are apparently harmless. The frequency of anti-PF4 was 5.6% and 8.0% after COVID-19 vaccination with Comirnaty (Pfizer/Biontech vaccine) and Vaxzevria (AstraZeneca), respectively [14]. These antibodies were usually of low concentration (OD 0.5–1.0) and did not aggregate platelets. The pathogenic features of sera from VITT/TTS patients are unique in their capacity to evoke healthy donor platelet activation and aggregation [15]. 

In our series, we observed that these pathogenic features of anti-PF4 antibodies in VITT were rapidly lost after initiating therapy, although they were still high in the ELISA. Platelet binding and aggregation appears to be a feature of immune complexes consisting of anti-PF4 antibodies and their target(s), i.e., the PF4 protein itself and the polyanionic substances that it binds to create macromolecular complexes. Accordingly, platelet binding and aggregation can be increased by adding external PF4 or the AZD1222 adenoviral particle that consists of a polyanionic capsid protein. The disappearance of platelet binding in our flow cytometric assay (where we add AZD1222 but not PF4) and aggregation in our modified HIPA tests (where we add buffer alone, AZD1222, or heparin) suggests that perhaps the release of PF4 is reduced after starting effective therapy and generation of immune complexes is reduced. Alternatively, or in addition, anticoagulants could also directly contribute to immune complex dissociation, as recently suggested [16].

Platelet binding and aggregation of patient Ig are abrogated by blocking the platelet FcγRIIA receptor with specific antibodies or IVIG [3], providing a rationale for IVIG treatment of VITT. However, the VITT pathology was also reported to encompass complement activation [17] and formation of neutrophil extracellular traps (NET) [18]. Both could contribute to thromboembolic events and explain, in part, refractory cases with new or progressive clinical events despite anticoagulation and IVIG, as reported in our series and by others [19,20].

The sustained elevation of anti-PF4 antibodies may, however, not be harmless just because platelet binding and aggregation was no longer detectable. We noted that D-Dimer levels remained elevated for months in most patients despite anticoagulation. D-Dimer levels usually drop sharply during anticoagulation of deep vein thrombosis [21], and the sustained increase seen in our series may point towards a prolonged prothrombotic condition that may or may not be related to the presence of anti-PF4 antibodies.

Our decision to provide oral anticoagulation for at least 6 months was mainly based on clinical grounds, considering the severity of the thromboembolic events in VITT patients. However, we felt encouraged to continue anticoagulation, seeing high D-Dimer as a potential sign of an ongoing prothrombotic condition. Likewise, we felt encouraged to stop anticoagulation earlier in two patients with less pronounced D-Dimer elevation and full clinical recovery. Such withdrawals did not result in relapse of thrombocytopenia or thromboembolic events.

Our results are in line with observations from the UK and international collaborations [22,23]. VITT/TTS is a rare syndrome with an estimated incidence of 1 case out of 150,000 AZD1222 vaccinations [24]. It is interesting to note that eight out of nine cases occurred after the first AZD1222 administration, very similar to previous reports. If a rare complication is mainly confined to the first of two administrations of an agent, this may suggest a high risk in very few people rather than a low risk in many. Until now, the root cause for this risk remains unclear.

The limitations of our study should not go unnoticed. Owing to its retrospective nature, we cannot draw firm conclusions about the efficacy of any therapeutic intervention. This is particularly relevant for the efficacy of corticosteroids, IVIG, and eculizumab. However, prospective studies will not be feasible in this disorder; therefore, accumulating retrospective reports of treatment efficacy and safety will likely constitute the only source of evidence in the foreseeable future.

## Figures and Tables

**Table 1:** Aggregated baseline data at time of hospital admission.

Category	Characteristic	Data
Demographics	Female gender, *n* (%)	8 (89)
	Age in years, mean (SD)	56.7 (12.4)
Diagnosis of VITT	Days after vaccination, mean (SD)	12 (5)
	Received Vaxzevria, *n* (%)	9 (100)
	Presenting after 1st vaccination, *n* (%)	8 (89)
	Presenting after 2nd vaccination, *n* (%)	1 (11)
Clinical	Signs or symptoms of thromboembolic event, *n* (%)	9 (100)
	Confirmation of thromboembolic event, *n* (%)	8 (89)
	Petechiae, *n* (%)	6 (67)
Laboratory data	Positive anti-PF4 antibodies (ELISA), *n* (%)	9 (100)
	Positive HIPA (with AZD1222), *n* (%)	7 (78)
	Platelet count per nL, median (range)	27 (12–105)
	D-Dimer in mg/L, median (range)	33.9 (18.4–>35)

**Table 2:** Details on thromboembolic events, initial anticoagulation, and VITT-specific therapy.

Patient Number	Clinical Events	Argatroban	Corticosteroids	IVIG	Eculizumab
1	Cerebral venous sinus thrombosis, thrombotic microangiopathy	− ^1^	+	−	+ (1st)
2	Arterial cerebral embolism	+	+	+	−
3	Transitory ischemic attack	+	+	−	−
4	Splanchnic vein thrombosis	+ ^2^	−	+ (1st)	+ (2nd)
5	Cerebral and popliteal artery thrombosis, DVT, pulmonary embolism	+	−	+	−
6	DVT	+	+	+ (1st)	+ (2nd)
7	None	+	+	−	−
8	Portal vein thrombosis, DVT	+	+	−	−
9	DVT	+	−	−	−

^1^ Patient received full-dose unfractionated heparin. ^2^ Patient also received alteplase. Abbreviations: 1st, first-line treatment; 2nd, second-line treatment; DVT, deep vein thrombosis; IVIG, intravenous immunoglobulin.

## Data Availability

Data are provided upon request by the corresponding authors.
